# Analysis of Urogenital Toileting Techniques and Their Association with Bacteriuria Rates Among Nursing Home Residents

**DOI:** 10.3390/microorganisms14071490

**Published:** 2026-07-08

**Authors:** Patricia Cuiña Iglesias, Dominique Correia De Oliveira, Marie Immaculée Nahimana Tessemo, Marie-Catherine Snoussi, Emmanouil Glampedakis

**Affiliations:** 1 Cantonal Unit for Infection Prevention and Control, Public Health Service, 1003 Lausanne, Switzerland; patricia.cuinaiglesias@hopitalvs.ch (P.C.I.); marie.nahimana-tessemo@vd.ch (M.I.N.T.); mc.snoussi-pirotte@vd.ch (M.-C.S.); 2 Haute Ecole de Santé Vaud (HESAV), Haute Ecole Spécialisée de Suisse Occidentale (HES-SO), 1011 Lausanne, Switzerland; dominique.correiadeoliveira@hesav.ch

**Keywords:** infection prevention and control (IPC), nursing homes, long-term care facilities (LTCF), Hygiene, Urogenital Toileting, urinary tract infection (UTI), infection, Bacteriuria

## Abstract

Urogenital toileting practices for residents in nursing homes (NHs) and their implications for the occurrence of bacteriuria remain largely unknown. Our purpose was to assess urogenital toileting practices in NHs in our region and their association with bacteriuria and urinary culture contamination rates. NHs reported on the direction of urogenital cleaning with respect to the urinary meatus and related practices during resident care over 7 years (2017 to 2023). Linear mixed models were used to relate two urogenital cleaning techniques, with bacteriuria rates (positive urinary cultures per resident over a year) and urinary culture contamination proportions. Of the 111 participating NHs, 33% used a technique involving outward cleaning with respect to the urinary meatus. Of these, 50 institutions had 7-year urinary microbiological data available (10,602 cultures, 13,059 isolates). *Escherichia coli* (45%) was the most frequently isolated microorganism while 12.4% of all cultures were contaminated (>3 microorganisms). We did not observe any significant association between the cleaning technique and the bacteriuria rates. The technique involving outward cleaning away from the urinary meatus was associated with lower odds of urinary culture contamination (odds ratio = 0.15, 95% CI: 0.05–0.48, *p* < 0.01). Urogenital cleaning techniques varied across participating institutions. We observed an association with urinary culture contamination, but not with bacteriuria rates. These findings warrant further investigation.

## 1. Introduction

The continuous aging of the population in the Organization for Economic Co-operation and Development (OECD) countries [1] implies that an increasing proportion of individuals will require residential living, assisted care, and highly skilled nursing services, particularly after the age of 65, which are frequently provided in nursing homes (NHs). Residents of NHs are especially susceptible to infections due to advanced age and overall frailty [2]. Healthcare-associated infections (HAIs) have significant consequences for residents, including reduced quality of life, the need for transfer to acute-care facilities, and death [3]. The mortality burden associated with HAIs in this vulnerable population remains substantial, even in the post-pandemic era [4].

The burden of HAIs is commonly assessed through point-prevalence surveys (PPSs), which use standardized methodologies to ensure comparability across time and geographic regions. The most recent European PPS conducted in 2023–2024, involving 18 EU/EEA countries, reported an overall HAI prevalence of 3.1% in NHs [5]. Similarly, in Switzerland, the most recent nationwide PPS across 94 NHs reported an HAI prevalence of 2.2% [6]. Although these rates may appear relatively low, it should be emphasized that PPSs provide only a snapshot of the overall HAI burden due to their cross-sectional design. In contrast, longitudinal prospective studies indicate that approximately 57% of NH residents experience at least one HAI over the course of a year [2]. Among these infections, 4.3% result in hospitalization and 4.5% are associated with death [2]. Urinary tract infections (UTIs) consistently rank among the most common HAIs in NHs, typically occupying the first or second position, and, depending on study design, account for approximately 18.7% to 46% of all NH-reported HAIs [2,5,6].

In parallel, antimicrobial resistance has emerged as a growing concern in the long-term care setting. Several studies have demonstrated that urinary bacterial isolates from NH residents tend to exhibit higher levels of antimicrobial resistance than those identified in outpatient populations [7,8,9], and increasing resistance rates have been reported over time [10,11]. As a result, NHs have become priority targets for antimicrobial stewardship initiatives. Given their high frequency among NH residents, the prevention of UTIs represents a priority focus for such interventions. However, most studies in this setting have primarily concentrated on urinary catheter use and the prevention of catheter-associated UTIs.

Studies conducted in younger, predominantly female populations have suggested that urogenital hygiene practices may influence the occurrence of UTIs [12,13,14,15,16,17,18]. In particular, the recommendation to wipe from front to back is widely included in preventive guidance for UTIs [19,20,21,22,23]. Urogenital hygiene may be especially important in NH settings, where residents require toileting assistance due to high rates of urinary and/or fecal incontinence [5,6], conditions that also lead to prolonged contact of urine and fecal material with the urogenital area potentially increasing the risk of UTIs [6]. Additionally, medical equipment used for the toileting of residents has been implicated in the transmission of microorganisms, including multidrug-resistant organisms, highlighting the importance of such nursing activities for residents [24,25,26].

Little is known about the relationship between urogenital toileting practices and their influence on the rate of bacteriuria among NH residents. While bacteriuria is common in this population, it is usually asymptomatic and only rarely indicates a true UTI [27]. Additionally, previous research has shown that treating asymptomatic bacteriuria provides no benefit and may even be harmful [28]. As a result, current guidelines do not recommend screening for or treating asymptomatic bacteriuria in this population [21]. Nevertheless, asymptomatic bacteriuria continues to be overdiagnosed and unnecessarily treated in this setting [29]. As a result, it contributes substantially to inappropriate antimicrobial use and antimicrobial resistance. Therefore, identifying factors associated with its occurrence may represent a valuable antimicrobial stewardship strategy for NHs.

The aim of this study was to provide an overview of the urogenital toileting techniques used in the care of NH residents across institutions of canton Vaud in Switzerland. In addition, we aimed to examine potential associations between toileting techniques and the frequency of bacteriuria, as well as the contamination rate of urinary cultures in participating institutions.

## 2. Materials and Methods

### 2.1. Setting

We included NHs that provide care for residents aged 65 years and older. The study was conducted in canton Vaud, one of the largest cantons in Switzerland. According to the cantonal information platform on health service utilization, as of 2023, Vaud has 120 NHs accommodating 6782 residents (mean age 85.5 years, 32% males, 68% females) [30]. NHs in canton Vaud operate as private non-profit and private for-profit institutions. Based on the profiles of their residents, these institutions are classified as geriatric (caring for residents with age-related physical dependency), psychogeriatric (for residents with neurodegenerative-related psychiatric conditions) and mixed institutions (providing care for residents with both types of needs). Institutions are also classified based on their location as urban (situated within towns and cities), intermediate (located on the outskirts of towns and cities), and rural. Medical care in the canton’s NHs is provided either by residents’ general practitioners or by attending physicians employed by the institution. Nursing care, including urogenital hygiene, is delivered by nurses and nursing assistants or trainees in these professions, collectively referred to as healthcare workers (HCWs). HCWs in the canton have free access to infection prevention and control (IPC) guidelines [31], as well as NH-specific guidelines for infection treatment [32], provided by the cantonal infection prevention and control unit of Vaud (HPCi Vaud). However, a standardized protocol for urogenital hygiene and cleaning is currently lacking. HPCi Vaud has established a robust IPC network within NHs, comprising IPC-trained link nurses. As a result, approximately 80% of NHs in the canton have a designated IPC link nurse. HPCi Vaud supports these facilities by promoting best practices, providing IPC education, monitoring IPC indicators, and conducting audits to ensure safe care for residents and the protection of HCWs.

### 2.2. Study Design and Temporal Extent

This was a retrospective epidemiological investigation employing an ecological study design based on aggregated data at the NH level. We conducted a survey aiming to investigate the urogenital cleaning techniques used in NHs between 2017 and 2023. All NHs in the canton were invited to participate. Invitations were sent via emails to NH directors, chief nurses, and, when available, IPC link nurses. A Microsoft Forms^®^ questionnaire was used for response data collection. The invitations were distributed in September 2023, allowing a flexible participation period, with final responses received in March 2024. Participation in the survey was voluntary.

### 2.3. Questionnaire

The questionnaire included the following items: the number of residents at the time of the survey, the name of the institution, location, facility type, the technique used for urogenital cleaning, the number of cleaning mitts used, and the change of water and reusable bath basin used during the cleaning procedure. The questionnaire also surveyed changes in the above-mentioned urogenital cleaning-related items between 2017 and 2023. The questionnaire was administered in French, the official language of the canton. An English version of the survey tool is provided in the Supplementary Materials.

### 2.4. Classification of Routine Daily Urogenital Cleaning Techniques

The majority of NH residents in our region require daily assistance with urogenital hygiene due to advanced age and the high prevalence of factors such as immobility, disorientation, cognitive impairment and/or incontinence [6,33]. Urogenital cleaning in NHs is performed using a wash mitt, a cloth glove impregnated with warm tap water and liquid soap, along with a reusable bath basin containing water and soap for rinsing and reapplying the cleaning solution. As there is no standardized local protocol for daily urogenital hygiene, we hypothesized that cleaning techniques may vary, particularly in terms of cleaning direction, which could influence the risk of contamination and subsequent bacteriuria.

Consequently, we classified urogenital hygiene practices based on the technique used. Technique A involved cleaning from the urinary meatus outward, a method hypothesized to reduce the risk of bacteriuria and urinary culture contamination. Technique B entailed cleaning from the pubis, inner thighs, and labia majora (females) / scrotum (males) toward the urinary meatus, which may introduce microorganisms into the urethral opening, and is thus hypothesized to increase the risk of bacteriuria and urinary culture contamination. However, if HCWs changed wash mitts, water, and the reusable bath basin between cleaning the outer and inner areas, the practice was still classified as Technique A in the study, even if the direction was inward.

### 2.5. Urine Specimen Collection During the Study Period (2017–2023)

The cantonal protocol for urinary sampling requires HCWs to follow specific measures to minimize the risk of culture contamination. In brief, HCWs must wear a face mask and a medical gown, disinfect their hands with an alcohol-based handrub, and put on non-sterile medical gloves. Urogenital cleaning is then performed with water and soap. Midstream urine is then collected for culture.

For residents with an indwelling urinary catheter, HCWs must wear a gown and face mask, disinfect their hands, and put on non-sterile medical gloves. Urine is collected from the dedicated sampling port of the catheter using a syringe.

Samples are immediately transferred to the laboratory for analysis or, if delayed, stored in accordance with the specimen collection fabricant indications.

### 2.6. Urinary Culture Database

We utilized the urinary culture dataset from HPCi Vaud, which includes anonymized results from positive urinary cultures of NH residents. The dataset records the annual number of positive urinary cultures and of isolated microorganisms in each participating NH overall and by microorganism category. For cultures positive with up to 3 isolates, each of them is counted in the corresponding microorganism category. Cultures positive with more than 3 isolates are classified as “contaminated” without consideration (counting) of the implicated microorganisms in specific categories. The clinical rationale for urinary sampling is unknown, and sterile urinary cultures are not included in the dataset.

### 2.7. Statistical Analysis

Findings related to categorical variables are presented as numbers and percentages while from continuous variables as means and standard deviations (SD).

Two outcome variables were analyzed: (i) the culture-to-resident ratio, calculated as the number of positive urinary cultures divided by the number of residents, representing the annual bacteriuria rate in each NH, and (ii) the contamination proportion, calculated as the number of contaminated cultures divided by the annual number of positive cultures in each NH. For the bacteriuria rate, cultures positive for *Candida* spp. or non-identifiable microorganisms (45 cultures, <0.5% of all cultures) were excluded to restrict the outcome to bacterial cultures only.

To relate urogenital cleaning techniques with the outcome variables we used two-level linear regression models accounting for the clustering of repeated measures in NHs (random intercept model). Only NHs with complete microbiological data between 2017 and 2023 were included in this analysis. The bacteriuria rate was log-transformed, and the contamination proportion was logit-transformed. Both models were adjusted for the number of residents, facility type, and NH location. Technique B was the reference category in these models.

Results of the fixed effects are presented as exponentiated β-coefficients with corresponding 95% confidence intervals (CIs). For the bacteriuria rate, the exponentiated β-coefficient represents the expected multiplicative change in the mean bacteriuria rate, whereas for urinary culture contamination it represents the corresponding odds ratio, associated with a one-unit change in the predictor variable (i.e., changing from Technique B to Technique A).

Results were statistically significant at *p* < 0.05. All analyses were performed using R Statistical Software (version 4.1.2; R Foundation for Statistical Computing, Vienna, Austria) on RStudio [34].

### 2.8. Ethical Statement

This study did not involve the collection of personal data from HCWs or residents and was conducted in accordance with the local ethic committee’s criteria on quality studies [35]; hence an ethics committee approval was not required.

## 3. Results

### 3.1. Participation

A total of 111 NHs participated in the urogenital hygiene practices survey, representing 92.5% of NHs in the canton. Among these, 50 NHs (45% of the participating NHs) had complete seven-year urinary culture data available (50/120, 42%).

### 3.2. Institutional Characteristics (All Participating Institutions)

Of the 111 participating NHs, 66 (59%) were located in urban, 24 (22%) in intermediate, and 21 (19%) in rural areas. Fifty-six NHs (50%) were geriatric, 30 (27%) were mixed, 24 (22%) were psychogeriatric facilities, and 1 (1%) specialized in elderly residents with neuromuscular disorders. The mean (SD) number of occupied beds was 54.1 (27.5), ranging from 10 to 163.

### 3.3. Urogenital Cleaning Practices (All Participating Institutions)

At the end of 2023, among the 111 participating NHs, 37 (33%) adhered to Technique A for urogenital hygiene. Of these, 34 institutions used an outward cleaning direction, while 3 used an inward direction but ensured changing wash mitts, reusable bath basins, and water between cleaning the outer and inner areas. Regarding the number of wash mitts used per procedure, 87 NHs reported using a single mitt, 16 used two mitts, and 8 used more than two mitts during the cleaning process. Only 21 institutions reported changing the water in the bath basin during the procedure, and of these, only 10 simultaneously replaced it with a clean basin. Five institutions reported using at least two wash mitts, changing the water and bath basin after each mitt change. Table 1 summarizes urogenital hygiene practices over time. There has been a slight increase in the number of institutions using Technique A over time (from 30/111, 27% to 37/111, 33%). Among the seven institutions that transitioned to Technique A during the study period, six changed cleaning direction from inward to outward, while one kept the inward direction but changing wash mitts, water, and the bath basin between cleaning outer and inner areas.

### 3.4. Institutional Characteristics (Institutions with Complete Microbiological Data)

Among the 50 institutions with complete seven-year microbiological data, 33 (66%) were located in urban, 12 (24%) in intermediate, and 5 (10%) in rural areas. Twenty-seven (54%) NHs were geriatric, sixteen (32%) mixed, and seven (14%) psychogeriatric-type facilities. Table 2 summarizes the number of residents hosted in these institutions over time, with a mean ranging from 57.5 to 64.9 residents during the study period.

### 3.5. Microbiological Characteristics (Institutions with Complete Microbiological Data)

**Figure 1:**
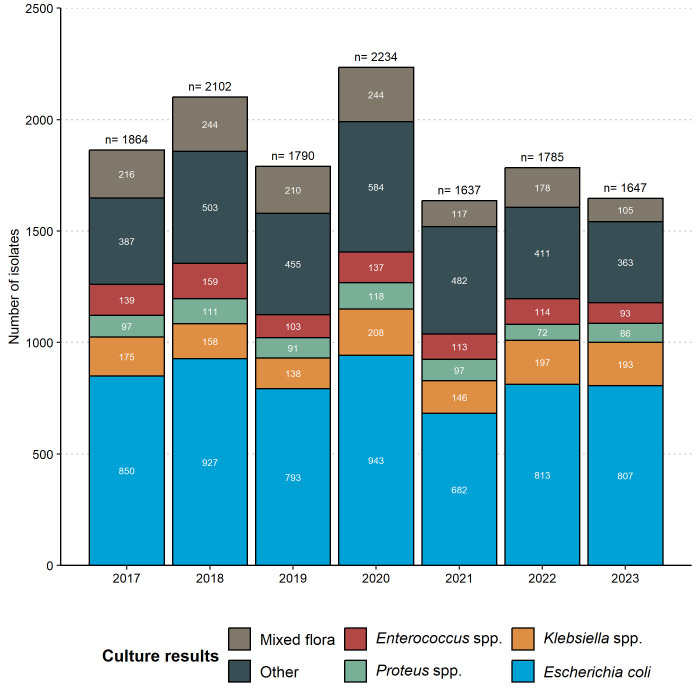
Summary of urinary microbiological results in institutions with complete microbiological data (N = 50). A total of 13,059 isolates from 10,602 positive urinary cultures. Mixed flora (n = 1314): cultures positive for > 3 microorganisms. Other microorganisms (n = 3185) included: *Streptococcus* spp. (n = 1042), *Staphylococcus* spp. (n = 469), other Gram-positive cocci (n = 412), *Pseudomonas* spp. (n = 411), *Enterobacter* spp. (n = 283), other enterobacteria (n = 264), other Gram-positive bacteria (n = 193), *Candida* spp. (n = 42), other Gram-negative non-fermenters (n = 41), *Serratia* spp. (n = 22), anaerobic bacteria (n = 3), and non-identifiable microorganisms (n = 3). spp.: species.

Over the seven-year period, a total of 10,602 positive urinary cultures, corresponding to 13,059 isolates, were recorded in the 50 NHs with complete microbiological data. *Escherichia coli* (5815, 45%) was the most frequently isolated microorganism, followed by *Klebsiella* spp. (1215, 9.3%). More detailed microbiological results over the study period are presented in Figure 1. The mean number of positive urinary cultures ranged from 29.3 to 35.4, with the mean positive culture rate ranging from 0.45 to 0.63 across the seven years. Overall, 12.4% of urinary cultures were classified as contaminated, with the mean contamination rate varying between 10.4% and 13.6%, as shown in Table 2. Table 3 illustrates the distribution of outcomes of interest during the study period. In terms of antimicrobial resistance, 711 *Escherichia coli* isolates were identified as extended-spectrum cephalosporin-resistant (ESC-R), corresponding to 12.2% of all *E. coli* isolates. ESC-R phenotypes were also detected in 41 *Klebsiella* spp. isolates (3.4%) and 8 *Proteus* spp. isolates (1.2%). Table 4 presents the annual distribution of ESC-R isolates within these three bacterial groups, as well as the overall ESC-R prevalence during the study period.

### 3.6. Urogenital Cleaning Practices (Institutions with Complete Microbiological Data)

At the end of 2023, among the 50 participating NHs with complete microbiological data, 15 (30%) adhered to Technique A. Of these, 14 institutions were using an outward cleaning direction, while 1 used an inward direction but ensured changing wash mitts, reusable bath basins, and water between cleaning the outer and inner areas. Regarding the number of wash mitts used per procedure, forty-three (86%) reported using a single mitt, three (6%) used two mitts, and four (8%) used more than two mitts during the cleaning process. Only six (12%) institutions reported changing the water in the bath basin during the procedure, and of these, only three were reported to have simultaneously changed to a clean bath basin. One institution reported using at least two wash mitts, changing the water and bath basin after each mitt change. Table 1 summarizes urogenital hygiene practices over time. There has been a slight increase in the number of institutions using Technique A over time (from 12/50, 24% to 15/50, 30%). All three institutions that reported changing their practice over time switched from an inward to an outward cleaning direction.

### 3.7. Urogenital Cleaning Practices and Outcomes of Interest

As demonstrated in Table 3, the mean bacteriuria rate ranged from 0.49 to 0.91 in NHs using Technique A and between 0.43 and 0.61 in those using Technique B. The mean percentage of contaminated cultures ranged from 3.14 to 6.92% in NHs using Technique A and between 12.3 and 16.4% in NHs using Technique B. The multilevel regression model did not reveal any significant association between the technique used and the bacteriuria rate (exp(β) = 1.27; 95% confidence interval (CI): 0.87–1.86; *p* = 0.22). Use of Technique A was associated with lower odds of urinary culture contamination (odds ratio = 0.15; 95% CI: 0.05–0.48; *p* < 0.01).

## 4. Discussion

In this multicenter study, we aimed to assess urogenital toileting techniques across NHs in our region and to evaluate their association with bacteriuria rates among residents. The results demonstrated variability in practices among participating institutions, with most facilities employing an inward direction during urogenital cleaning. No association was identified between cleaning techniques and bacteriuria rates. In institutions using an inward cleaning direction, a higher frequency of urinary culture contamination was observed.

Several previous studies have examined and found associations between cleaning practices, such as wiping direction and urogenital hygiene after defecation, and the risk of UTIs [12,13,14,15,16,17,18]. However, these studies were conducted predominantly in younger female populations, and their findings cannot be readily extrapolated to institutionalized, multimorbid populations such as NH residents. Our study included NHs that mainly host older female residents, among whom previous estimates have reported a urinary catheter prevalence of approximately 6% [5,6]. Additionally, the present study focused on bacteriuria rates and did not observe any association between toileting practices, particularly the direction of cleaning relative to the urinary meatus, and the frequency of bacteriuria. Nevertheless, these findings should be interpreted with caution given the retrospective observational design and the absence of resident-level data, which precludes causal inference at the individual level. Analyses based on aggregated data may have attenuated potential associations, and studies incorporating more granular resident-level information are needed to further investigate this relationship. Furthermore, our study included urinary cultures for which the clinical indication was unknown. Specifically, we could not determine whether cultures were obtained because of suspected UTI, inappropriate screening for asymptomatic bacteriuria, or non-localizing geriatric symptoms (e.g., falls or functional decline). Although screening for asymptomatic bacteriuria and urine testing for non-localizing geriatric symptoms are not recommended [36], including in our region [32], these practices remain common [29]. Consequently, the bacteriuria rate reported in this study should not be considered a measure of UTI incidence.

In contrast, we observed an association between an outward cleaning direction and a lower rate of urinary culture contamination. We hypothesize that this finding may be explained by a reduced risk of introducing polymicrobial fecal flora into the urinary tract during the toileting process. Such a reduction may also have important clinical implications, as polymicrobial contamination frequently necessitates repeat urine cultures and may delay the initiation of targeted antibiotic therapy. This may, in turn, increase healthcare costs and contribute to inappropriate antibiotic administration, with potential consequences including treatment failure and the development of antimicrobial resistance. Nonetheless, this finding should be interpreted with caution given the ecological study design, which does not permit conclusions at the individual resident level. This analysis was based on urinary cultures classified as mixed flora in our database, defined as cultures yielding more than three isolates. Therefore, the potential impact on cultures positive for only two or three microorganisms was not assessed. According to previous unpublished data from our unit, cultures positive for two microorganisms account for approximately 17% of all urinary cultures, whereas cultures positive for three microorganisms represent approximately 4%. As a result, our findings may not fully capture the association between urogenital toileting practices and less extensive polymicrobial growth.

Most participating NHs employed an inward direction during urogenital cleaning, and this practice remained largely unchanged over time. The variability in cleaning techniques observed across institutions could be explained by the absence of regional, national, or international guidelines addressing this common nursing practice in a population frequently affected by urinary and/or fecal incontinence.

A major strength of our study is that it addresses a largely underexplored aspect of nursing care practice across a large number of institutions caring for elderly residents. Although urinary and/or fecal incontinence is highly prevalent among NH residents [5,6] and constitutes a well-recognized risk factor for HAIs [6], particularly UTIs, practices related to urogenital hygiene and toileting care have received limited scientific attention in this population. By providing a multicenter overview of current practices, our study contributes important preliminary data in an area where evidence remains scarce and highlights substantial interinstitutional variability in routine nursing procedures. These findings may serve as a foundation for the development of future prospective and interventional studies aimed at identifying evidence-based best practices for urogenital care in institutionalized older adults.

In addition to the clinical implications of urogenital toileting, our study also explored several IPC components of toileting care, including the use of clean water, washing mitts, and wash basins during the cleaning process. These practices are clinically relevant, as inadequate handling or reuse of equipment intended for personal hygiene and toileting nursing acts have previously been associated with the risk of contamination and transmission of multidrug-resistant organisms [24,25,26]. The assessment of these complementary IPC measures broadens the scope of the study and underscores the complexity of urogenital care as a potential contributor to microbial transmission in NH environments. By incorporating these additional variables, our study provides a more comprehensive evaluation of routine toileting practices and identifies several domains that warrant further standardization and investigation.

In our study, the overall rate of ESC-R among *Escherichia coli*, *Klebsiella* spp., and *Proteus* spp. isolated across all participating institutions during the study period was 9.9%. Although our urinary culture database did not contain information on resistance profiles of other microorganisms, previous studies conducted in Swiss NHs have shown that ESC-R remains the predominant antimicrobial resistance concern in this setting. In contrast, other resistance profiles, such as carbapenem-resistant *Enterobacterales* and glycopeptide-resistant enterococci, are exceedingly rare, with reported prevalences of 0.3% and 0.4%, respectively [11]. Nevertheless, antimicrobial resistance epidemiology is dynamic and may evolve over time. Continued surveillance studies are therefore warranted to monitor trends in antimicrobial resistance and to detect emerging resistance mechanisms in these particularly vulnerable care environments.

Our study has several limitations that should be acknowledged. First, the retrospective observational and ecological design precludes any inference of causality between toileting practices and individual-level outcomes such as the risk of bacteriuria or culture contamination. Second, the analysis was performed at the institutional rather than resident level, limiting the ability to account for individual resident characteristics and potential confounding factors. Participating institutions reported their general care practices and employed cleaning principles; however, the actual technique applied by individual HCWs during routine care could not be verified. In addition, multiple HCWs may provide care to the same residents, potentially resulting in variability in nursing practices within a single institution or even for the same resident over time. Several important resident-level variables were unavailable, including sex, comorbidities, functional status, degree of urinary and/or fecal incontinence, and the presence of urinary catheters. These factors are clinically relevant, as age [37], female sex [37], incontinence [6], and catheterization [6,37] are all recognized risk factors for UTI, while sex, the presence of urinary catheters, and some comorbidities seem to influence the rates of asymptomatic bacteriuria [27]. Therefore, their consideration in analyses may have influenced the observed associations. Furthermore, urinary culture testing strategies likely varied across facilities. Differences in the thresholds for obtaining urine cultures may have influenced the observed bacteriuria rates independently of the true underlying burden of bacteriuria. Although physicians had access to harmonized guidelines [32] for the diagnosis and treatment of UTIs, it was not possible to distinguish between cultures obtained for symptomatic infection and those obtained for nonspecific clinical deterioration frequently observed in this population. This limitation is particularly important in elderly institutionalized populations, where asymptomatic bacteriuria is common [27] and urine cultures may not reliably reflect true infection. Consequently, urinary culture frequency should not be used as a surrogate marker for UTI.

## 5. Conclusions

In conclusion, this multicenter study highlights substantial variability in urogenital cleaning practices across NHs and underscores the absence of standardized guidance for this common nursing care procedure in a highly vulnerable population. It also highlights the need to revise vocational training curricula by providing an evidence-based foundation for the standardization of urogenital hygiene practice. Although no significant association was identified between cleaning techniques and the bacteriuria rates, we observed a lower frequency of urinary culture contamination in institutions using the outward cleaning direction, which should be further explored.

Given the high prevalence of urinary and fecal incontinence among institutionalized older adults, as well as the important clinical, IPC, and antimicrobial stewardship implications of contaminated urine cultures, optimizing urogenital hygiene practices may represent a simple and low-cost opportunity to improve infection prevention and quality of care in long-term care settings. Our findings provide an important foundation for future research and emphasize the need for prospective resident-level studies to better define evidence-based recommendations for urogenital toileting practices in NHs.

## Figures and Tables

**Table 1:** Summary of urogenital cleaning techniques over time among the institutions participating in the survey.

Characteristic	All NHs N = 111		Complete Microbiological Data NHs N = 50	
	2017–2019	2020–2023	2017–2019	2020–2023
	n (%)			
Technique A	30 (27%) ^1^	37 (33%) ^2^	12 (24%) ^3^	15 (30%) ^3^
Outward direction	28 (25%)	34 (31%)	11 (22%)	14 (28%)
Technique B	81 (73%)	74 (67%)	38 (76%)	35 (70%)
≥2 wash mitts	21 (19%)	24 (22%)	7 (14%)	7 (14%)
Water change	21 (19%)	21 (19%)	6 (12%)	6 (12%)
Reusable bath basin change	10 (9%)	10 (9%)	3 (6%)	3 (6%)

^1^ Inward direction, changing wash mitts, and using new water and cleaning basin (n = 2). ^2^ Inward direction, changing wash mitts, and using new water and cleaning basin (n = 3). ^3^ Inward direction, changing cleaning wash mitts, and using new water and cleaning basin (n = 1). n, N: number, NH: Nursing home.

**Table 2:** Characteristics of institutions with complete microbiological data (N = 50).

Year	Residents	Positive Cultures	Positive Cultures per Resident	Contaminated Cultures
	Mean (SD)			
2017	62.4 (31.1)	31.4 (37.7)	0.61 (0.94)	11.9% (16.7)
2018	63.1 (31.2)	34 (37.1)	0.63 (0.86)	11.7% (17)
2019	62.6 (31.3)	29.7 (26)	0.54 (0.55)	11.2% (17.2)
2020	62.7 (31.4)	35.4 (28.2)	0.63 (0.52)	13.6% (20.1)
2021	57.5 (28.9)	24.5 (20.6)	0.51 (0.55)	10.4% (17.6)
2022	60.9 (29.5)	29.3 (25.4)	0.57 (0.76)	10.4% (15.8)
2023	64.9 (32.6)	27.9 (21.8)	0.45 (0.31)	10.4% (21.1)

N: number; SD: standard deviation.

**Table 3:** Outcomes of interest by urogenital cleaning technique in NHs with complete microbiological data (N = 50).

Year	Bacteriuria Rate		Contaminated Cultures	
	Technique A	Technique B	Technique A	Technique B
	Mean (SD)			
2017	0.91 (1.68)	0.51 (0.52)	4.11% (9.25)	14.4% (17.9)
2018	0.84 (1.43)	0.56 (0.59)	3.14% (10.2)	14.4% (17.9)
2019	0.66 (0.87)	0.49 (0.41)	3.97% (9.37)	13.5% (18.5)
2020	0.65 (0.59)	0.61 (0.48)	6.92% (12.8)	16.4% (22.1)
2021	0.57 (0.58)	0.48 (0.53)	5.9% (15.3)	12.3% (18.4)
2022	0.83 (1.27)	0.45 (0.29)	5.15% (12.1)	12.6% (16.8)
2023	0.49 (0.25)	0.43 (0.34)	5.97% (16)	12.3% (22.8)

N: number; SD: standard deviation.

**Table 4:** Extended-spectrum cephalosporin resistance in NHs with complete microbiological data (N = 50).

Year	Extended-Spectrum Cephalosporin Resistance			
	*Escherichia coli*	*Klebsiella* spp.	*Proteus* spp.	Total
	% of Isolates			
2017	14.7%	10.3%	1%	12.8%
2018	13.7%	1.9%	0.9%	11%
2019	12.2%	0%	1.1%	9.6%
2020	11.6%	3.8%	1.7%	9.4%
2021	16.7%	1.4%	2.1%	12.8%
2022	8.9%	1%	0%	6.8%
2023	8.3%	4.1%	1.2%	7%
Overall (2017–2023)	12.2%	3.4%	1.2%	9.9%

N: number.

## Data Availability

The data presented in this study are available on request from the corresponding author due to privacy reasons.

## Supplementary Materials

The following supporting information can be downloaded at https://www.mdpi.com/article/10.3390/microorganisms14071490/s1, Questionnaire on Urogenital Toileting Practices.

## Abbreviations

The following abbreviations are used in this manuscript:

CI	Confidence interval
ESC-R	Extended-spectrum cephalosporin resistance
EU/EAA	European union/European economic area
HAI	Healthcare-associated infection
HCW	Healthcare worker
IPC	Infection prevention and control
NH	Nursing home
OECD	Organization for Economic Co-operation and Development
PPS	Point prevalence survey
SD	Standard deviation
UTI	Urinary tract infection
