# Supplementary material for: Analysis of Urogenital Toileting Techniques and Their Association with Bacteriuria Rates Among Nursing Home Residents
Source: Microorganisms. 2026 Jul 8;14(7):1490. doi: 10.3390/microorganisms14071490 (PMC13413991; doi:10.3390/microorganisms14071490)
Supplement: Supplementary file 1 [file microorganisms-14-01490-s001.zip › microorganisms-4391036-supplementary.pdf]

canton de vaud
DÉPARTEMENT DE LA SANTÉ
ET DE L'ACTION SOCIALE
Office du médecin cantonal

HPCi | hygiène, prévention et contrôle de l'infection

Questionnaire on Urogenital Toileting Practices

Institution:
Type of institution: □ Geriatric □ Psychogeriatric □ Mixed □ Other: …………….
Number of beds: ……………… **Number of residents:** ……………………
Localization: □ Urban □ Rural □ Intermediate

Questions

1. In which order do you perform urogenital hygiene care?

□ From the proximal area[1] to the distal area[2] □ From the distal area[2] to the proximal area[1]

2. How many wash mitts or disposable bathing mitts do you use during urogenital hygiene care?

□ One □ Two □ More than two: ……….

3. Do you change water between cleansing the proximal and distal areas?

□ Yes □ No

4. If yes, do you use a clean basin or reuse the same basin?

□ Reuse the same basin □ Use a clean basin

5. Have you modified your urogenital hygiene care technique within the past years (2017 to 2023)?

□ Yes □ No

If yes in Question 5: in which year and what change was implemented?

Year & change :
………………………………………………………………………………………………………
………………………………………………………………………………………………………

Additional comments

………………………………………………………………………………………………………
………………………………………………………………………………………………………

Definitions of Proximal and Distal Areas Relative to the Urethral Meatus

**Sex**	[1] **Proximal area** (closest to the urethral meatus)	[2] **Distal area** (further from the urethral meatus)
Female	Urethral meatus, labia minora, vaginal introitus	Inner thighs, inguinal folds, pubic region, labia majora
Male	Urethral meatus, glans penis	Inner thighs, inguinal folds, penile shaft, scrotum
